# Chitosan and β-Cyclodextrin-epichlorohydrin Polymer Composite Film as a Plant Healthcare Material for Carbendazim-Controlled Release to Protect Rape against *Sclerotinia sclerotiorum* (Lib.) de Bary

**DOI:** 10.3390/ma10040343

**Published:** 2017-03-26

**Authors:** Delong Wang, Mingchen Jia, Lanying Wang, Shuang Song, Juntao Feng, Xing Zhang

**Affiliations:** 1 Research and Development Center of Biorational Pesticide, Shaanxi Research Center of Biopesticide Engineering & Technology, Key Laboratory of Plant Protection Resources and Pest Management of Ministry of Education, Northwest A & F University, Yangling 712100, Shaanxi, China; rizhaoalong@163.com (D.W.); songshuang109@126.com (S.S.); zhangxing1952@hotmail.com (X.Z.); 2 Key Laboratory of Synthetic and Self-Assembly Chemistry for Organic Functional Molecules, Shanghai Institute of Organic Chemistry, Chinese Academy of Sciences, 345 Lingling Road, Shanghai 200032, China; jiamc@sioc.ac.cn; 3 College of Environment and Plant Protection, Hainan University, Haikou 570228, Hainan, China; daivemuwly@126.com

**Keywords:** β-cyclodextrin-epichlorohydrin, chitosan film, antifungal activity, controlled release, *Sclerotinia sclerotiorum* (Lib.) de Bary

## Abstract

The influence of β-cyclodextrin-epichlorohydrin (β-CD-EP) polymers on the improvement of the solubility and antifungal activity of carbendazim has been investigated. Meanwhile, the potential of the chitosan and β-CD-EP composite film used as a plant healthcare material for carbendazim-controlled release to protect rape against *Sclerotinia sclerotiorum* (Lib.) de Bary has been evaluated. β-CD-EP-1 and 2 (β-CD content, 750 mg/g and 440 mg/g, respectively) were found to significantly improve the solubility of the guest molecule carbendazim (17.9 and 18.5 times, respectively) and the 1:1 stoichiometry of the host-guest was confirmed by the Job’s plot. A slight synergism was observed for the β-CD-EP/carbendazim complex against *S. sclerotiorum* (Lib.) de Bary, indicating an enhancement to the bioavailability of carbendazim. The in vitro release studies revealed that β-CD-EP polymers could efficiently modulate carbendazim release behaviors, such as the release retard and rate. The in vivo efficacy experiments demonstrated that the β-CD-EP/carbendazim and chitosan composite film could significantly prolong the effective duration of carbendazim at a concentration of 100 μg/mL compared with spraying carbendazim at 500 μg/mL. Thereby, a highly useful and strategic concept in plant disease control by a plant healthcare material—the chitosan and polymeric β-CD-EP composite film—is provided, which could also serve as a concept for related plant diseases.

## 1. Introduction

Plants suffer from the emerging infectious diseases induced by phytopathogens, fungi, or fungal-like oomycetes, which have become increasingly aggravating worldwide [1,2,3]. Especially for staple crops, fruits, and vegetables, their attainable yields could be severely lessened as a consequence of the phytopathogenic infections. More importantly, many phytopathogens could spoil the postharvest products, leading to deteriorated quality, such as the fungus *Aspergillus flavus* that produces the mammal-lethal mycotoxic poison aflatoxin [4]. However, the global food demand, accompanied by the growing population and consumption, will maintain a lasting increase for at least 40 years [5]. Fortunately, since the 1960s a great variety of synthetic fungicides have been put into crop-protection use in agriculture and they have decreased crop losses while promoting yields and quality. For those fungicides before application, the technical materials in the concentrated state are always processed according to their physicochemical characteristics and are made as different formulations for application purposes, such as wettable powder, aqueous solution, microemulsion, and smoke [6]. As a result, the concentrated materials are dramatically dispersed when they are applied in fields by dusting, spraying, or fumigation. However, enhancing the dispersity usually brings about the intensified drift effect of fungicides, giving rise to great waste of the active ingredients with few amounts to target, and undesirable environmental toxicity [7,8,9]. The adverse drifting could be partially conquered by controlling the dispersity of the active ingredients through a controlled release system. To date, many controlled release formulations of pesticides have been developed to prolong the effective duration and reduce the undesirable toxicity, of which many have already lived up to commercial status [10].

In the pharmaceutical, cosmetics, and food industries, β-cyclodextrin (β-CD) either in solution or solid phase has been intensively focused on because of its cavity of suitable size which could non-covalently encapsulate guest molecules with special functionalities [11,12]. Owing to the formation of inclusion complexes, β-CD could improve the solubility, stability, and bioavailability of drugs, reduce the volatility of cosmetic perfumes, and mask unpleasant odors of functionalized molecules [13]. However, it is the low aqueous solubility and undesirable toxicity of β-CD that limit its wide applications in formulations [14]. These limitations could be eliminated as much as possible by using the hydrophilic and non-toxic β-CD polymers. Besides, it could be supposed that the three-dimensional polymeric networks could afford diverse binding sites to guest molecules, which thereby efficiently improve their apparent solubility [14,15,16]. Nevertheless, β-CD or its derivative-encapsulated formulations are presented mostly in a powdery state, and are short of manipulation in cases such as tissue engineering and wound dressing. Therefore, in many cases, they need a support for further processing. Chitosan, the deacetylated product of naturally abundant chitin, is a linear biomacromolecule. Its biodegradable and non-toxic profiles have made it intensively investigated by researchers [17,18]. More importantly, its good solubility in acid solutions meets the manufactured necessities of different application purposes, such as the drug-delivery chitosan sponges or films [19]. Thus, taking advantage of the chitosan matrix as a support, the hydrophilic β-CD polymers can be easily processed into desired forms to be used as controlled drug delivery systems.

**Figure 1:**
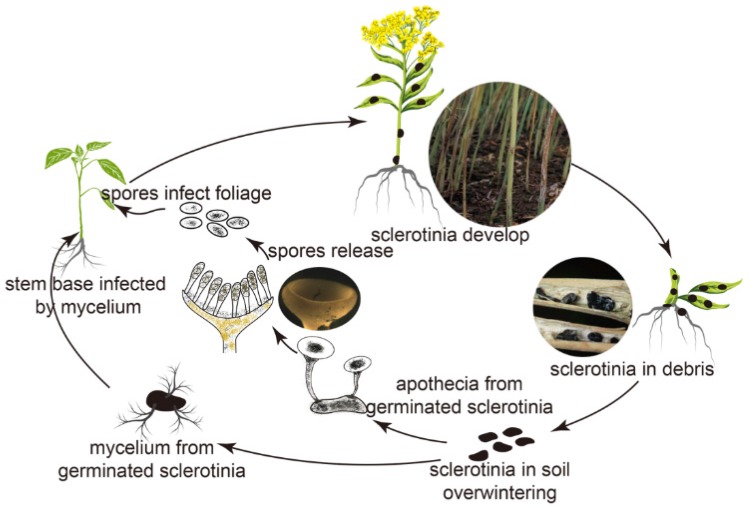
The disease cycle of sclerotinia stem rot in oilseed rape caused by *S. sclerotiorum* (Lib.) de Bary.

Rape (*Brassica napus* Linnaeus), an important industrial crop, matters to our daily life since its seeds are the raw material to produce edible oil, whose production accounts for 10%–15% of the worldwide food oil market share, and which furnishes animals with a high-protein feed. Due to the high oil yield of rape seed, many research centers in Europe have utilized its oil as the preliminary feedstock to produce biodiesel [20,21]. Sclerotinia stem rot, a devastating phyto-disease caused by *Sclerotinia sclerotiorum* (Lib.) de Bary in all main rape cultivation areas, could impact almost the whole growth stage of rape from seedling to maturation [22]. Particularly, the stem attack by *S. sclerotiorum* (Lib.) de Bary is the most severe infection, because this infection can cause the seedlings’ death and potential yield losses of 30% to 40% at the maturation stage [23]. It is common for the stem bases of the rape plant to be infected because hyphae originated from the germination of overwintering residual sclerotia in the soil can penetrate surrounding stem bases, resulting in serious basal stem infection, as can be illustrated from the disease cycle in Figure 1 [24]. Therefore, it is critical to block the stem infection from hyphae in the protection of seedlings and the decrease of yield losses.

An adhesive wound dressing usually comprises a semi-permeable polymeric membrane in which an adhesive side covers human skin [25,26,27]. The membrane matrix usually encapsulates different therapeutic drugs to control their release, which could efficiently reimburse the disadvantages associated with topical drugs and promote wound healing [28]. Inspired by the adhesive wound dressings and their functionalization with active drugs, we envisioned that the chitosan film composite with hydrophilic β-CD polymers could be designed as a renewable fungicide-controlled release system for blocking the rape stem infection from hyphae, which has not yet been extensively studied. To this end, we first prepared the water-soluble β-CD-epichlorohydrin (β-CD-EP) polymers and investigated their interactions with carbendazim, a systemic broad-spectrum fungicide used widely to efficiently control sclerotinia stem rot. To the best of our knowledge, the influence of β-CD-EP polymers on the improvement of the solubility and antifungal activity of carbendazim has not been reported before. Meanwhile, ours is the first study to explore the potential of the chitosan and β-CD-EP composite film used as a plant healthcare material for fungicide-controlled release. Herein, the controlled release profiles of the chitosan and β-CD-EP/carbendazim complex composite film were evaluated in vitro and in vivo. 

## 2. Materials and Methods

### 2.1. Materials

Commercial grade chitosan, which has a degree of deacetylation of 85.6% determined by hydrogen bromide titrimetric analysis [29] and a viscosimetric molar weight of 106 kDa determined by viscosity measurements [30] was purchased from Sinopharm Chemical Reagent Co., Ltd (Shanghai, China). Commercial grade β-cyclodextrin (β-CD) (molecular weight, 1134.98 g/mol) with purity ≥98% and epichlorohydrin (EP) with purity ≥98% were also purchased from Sinopharm Chemical Reagent Co., Ltd. Carbendazim (purity >98%) was purchased from Shanghai Aladdin Reagent Co., Ltd. (Shanghai, China) and was used without further purification. Other chemicals used were of analytical reagent grade. The redistilled water was used throughout. *S. sclerotiorum* (Lib.) de Bary was provided by the Research and Development Center of Biorational Pesticide, Northwest A & F University. 

### 2.2. Syntheses and Characterizations of β-CD-EP Polymers

#### 2.2.1. Syntheses of β-CD-EP Polymers

β-CD-EP polymers were prepared according to a previous report in the literature [31]. Generally, a homogeneous mixture of 5.67 g β-CD and 10 mL NaOH aqueous solution (33 wt %) was first obtained after 24 h-stirring at 25 °C. To the above solution, different amounts of epichlorohydrin (EP) (Table 1) were added rapidly and stirred vigorously with a magnetic stirrer for 4–8 h at 35 °C. Finally, the polymerization reaction was stopped by the addition of acetone. After decantation of the acetone, the obtained solution was kept at 55 °C overnight and then cooled to room temperature. After adjusting the pH to 7.0 using 6 M HCl, the solution was dialysed (molecular weight cut off, 1000). The residual solution was evaporated and triturated by adding ethanol. The pulverized powder was dried and kept in a vacuum desiccator in darkness before use.

#### 2.2.2. Characterizations of β-CD-EP Polymers

NMR spectroscopy. Samples β-CD and β-CD-EP were dissolved in deuteriumoxide (D_2_O) and recorded with their ^1^H NMR spectra at 25 °C using a 500 MHz Bruker Avance spectrometer. Chemical shifts (δ) are adjusted based on water (δ = 4.8).

FT-IR spectroscopy. The Fourier transform infrared spectra of all the solid samples were recorded on a Thermo Nicolet 380 (Thermo Nicolet Company). Samples in KBr pellets were scanned at a resolution of 4 cm^−1^ and 32 scans per spectrum, from 4000 to 400 cm^−1^.

Thermogravimetric analysis (TGA). TGA of β-CD and β-CD-EP polymers was performed on a TA Q500 thermal analysis system. Each sample was put into an aluminum pan under a constant N_2_ flow. The temperature was set up by a programmer, running from 25 to 800 °C at the rate of 10 °C/min. Each test was done in triplicate.

Dynamic light scattering (DLS). The apparent average hydrodynamic diameters of β-CD-EP polymers in distilled water at a concentration of 5 mg/mL were determined by DLS (Zetasizer Nano ZS-90, Malvern Instruments). A 633 nm laser source and a detection angle of 90° were used. The value was recorded as the average of three measurements.

Solubility. A saturated solution was prepared by addition of the 0.5 g sample to 1.0 mL of distilled water. After shaking mechanically for 4 h, the solutions were left standing overnight at room temperature. After filtration of the overnight solutions through 0.22 μm pore size syringe filters, the filtrate was dried until a constant weight was achieved. The solubility was estimated by the weight of the dried filtrate and was expressed in g/100 mL. Three repeats were conducted.

### 2.3. Preparation of β-CD-EP/Carbendazim Complexes

The kneading method by adopting the reported procedures was used to prepare the β-CD-EP/carbendazim complexes [32,33,34]. To the mixture of carbendazim and β-CD-EP with equal molar quantities, an appropriate amount of hydro-alcoholic solution (9/1, v/v) was added at regular intervals to maintain suitable consistency. Then the thick mixture was manually ground for 60 min. Afterward, the obtained paste was dried in a vacuum oven at 37 °C for 24 h and then washed with adequate acetone. Finally, the complex was dried again and pulverized into powder. The drug loading efficiencies (LE) were assayed (Table 2). Briefly, the proper amounts of complexes were dissolved in excessive distilled water, and they were submitted to spectrofluorimetric analysis after a complete dissolution. The LE is defined as Equation (1):

(1) $$\mathrm{LE}=\frac{W_{d}}{W_{t}}\times 100\%$$

where *W_d_* and *W_t_* represent the quantities of the drug in the complex and the total complex, respectively.

### 2.4. Interactions between β-CD-EPs and Carbendazim in Solution

#### 2.4.1. Spectrofluorimetric Analysis of Carbendazim

All the spectrofluorimetric analyses were performed at room temperature with a Hitachi-F4500 fluorescence spectrophotometer (Hitachi Ltd., Tokyo, Japan) interfaced with a microcomputer and processed by FL-solution software. Standard quartz fluorescence with 1-cm path lengths were used for measurements and an Eppendorf pipette of 20–200 μL (Germany) was used for dilutions. After addition of an aliquot of carbendazim aqueous solution into the quartz cuvette cell, the spectrofluorimetric analysis was run at a scanning speed of 200 nm/min. The fluorescence intensity was recorded at fixed maximum wavelengths of carbendazim in excitation (λ_ex_ = 285 nm) and emission (λ_em_ = 320 nm). The distilled water signal as the blank was used to correct all fluorescence measurements.

#### 2.4.2. Phase Solubility Studies of β-CD-EP/Carbendazim Complexes

Phase solubility studies were carried out following the reported method [35]. To screw-capped vials containing 5 mL of aqueous solution of β-CD ranging from 0 to 50 mmol/L were added an excess amount of carbendazim. The amount of β-CD-EP polymers was added according to the conversion of the β-CD content in the polymer. Each vial enclosed with tin foil was shaken at 28 °C until equilibrium was reached. After filtration of the mixture with a 0.22 μm pore size membrane, the filtrate was diluted for spectrofluorimetric analysis. Control samples were prepared without the addition of the test compound in the same fashion and were used as the zero setting. The standard curve was developed from the absorbance of 5 different standard concentrations in aqueous solution. All studies were carried out in triplicate. The stability constants, *K_S_* (L/mol), were calculated by Equation (2).

(2) $$K_{S}\;=\;\frac{\mathrm{slope}}{S_{0}\left(1-\mathrm{slope}\right)}$$

where *S*_0_ (mol/L) is the intercept (solubility in water without β-CD).

#### 2.4.3. Determination of Complex Stoichiometry

The continuous variation (Job’s plot) method was employed since it could provide a reliable confirmation of the complex stoichiometry [36,37]. An equal molar concentration of carbendazim and β-CD-EP polymer aqueous solutions (0.6 mmol/L) were used for mixing, in which the total molar concentration was kept as a constant (i.e., [carbendazim] + [β-CD-EP] = M), while the molar fraction of carbendazim (*r* = [carbendazim]/([carbendazim] + [β-CD-EP])) varied from 0.1 to 0.9. The mixture was mechanically shaken for at least 20 min to achieve the formation of inclusion complexes. In order to determine the stoichiometry, the fluorescence emission intensity variations (△*F*) of carbendazim were plotted against *r*.

### 2.5. The In Vitro Antifungal Effects of β-CD-EP/Carbendazim Complexes

The antifungal activities of carbendazim and the β-CD-EP/carbendazim complex against *S. sclerotiorum* (Lib.) de Bary in vitro were evaluated according to the mycelial growth inhibition assay reported previously [38]. Stock solutions with five different concentration gradients were prepared by dissolving carbendazim and the complex individually in sterilized water. The stock solutions (1 mL) were added rapidly to thawed potato dextrose agar (PDA) culture medium (9 mL) under 50 °C and they were mixed homogeneously before pouring into Petri dishes. The concentration of carbendazim in the final solutions were 0.025, 0.0625, 0.125, 0.20, and 0.40 μg/mL, respectively. After solidification, the plates were incubated with a 0.5 mm mycelium disk, inverted, and cultured at 25 °C for 48 h. The medium with β-CD-EP was used as the blank control. Three replicates of each test were carried out. The mycelial growth diameter (mm) was the average of the data measured crosswise after 48 h of culture. The growth inhibition rates (*I*) were calculated following the equation below:

(3) $$I=\frac{C-T}{C}\times 100\%$$

where *I* is the growth inhibition rate (%), *C* is the control group diameter (mm), and *T* is the treatment group diameter (mm). After conversion of the inhibition rates into probability values, the growth inhibition probability values were plotted against the logarithm of the concentration of carbendazim and the toxicity regression equations were thus established. The EC_50_ values (the concentration to offer 50% inhibition) of pure carbendazim and the complex were calculated from the established equations.

### 2.6. Preparation and Characterization of the Chitosan and β-CD-EP/Carbendazim Composite Films

#### 2.6.1. Preparation of Chitosan and β-CD-EP/Carbendazim Composite Films

The β-CD-EP/carbendazim complexes were obtained following the kneading procedure (see Section 2.3). The coating solutions (2% w/v) were prepared by dissolving chitosan in acetic acid (1% v/v). The prepared complexes were added to the chitosan solutions and the resulting mixture was stirred vigorously for 5 min to obtain a homogeneous solution. The loading concentration of carbendazim in the solution was 100 μg/mL. Afterwards, 500 μL solutions were withdrawn and coated uniformly on a micro cover glass (Matsunami glass IND., LTD., Kishiwada, Japan). The films were dried for 48 h at room temperature before further characterization. In the case of the β-CD-EP alone, the film was obtained in the same way. The chitosan film without the complexes or β-CD-EP was used as the control.

#### 2.6.2. Characterization of the Composite Films

Contact angles were measured in static mode using a XG-CAMB1 contact angle meter (Shanghai Xuanyichuangxi Industrial Equipment Co., Ltd., Shanghai, China). Onto the above prepared films was deposited a drop of 10 μL of distilled water by a 22-gauge needle. All the contact angle values were measured at 5 different positions on the prepared films. Each angle value was obtained from the imaging software supplied.

The surface morphology of the chitosan films was examined by field emission scanning electron microscopy to detect the changes in the surfaces before and after composition (FE-SEM, Hitachi S-4800, Hitachi Ltd., Tokyo, Japan). The prepared films were fixed on a brass stub using double-sided adhesive tape and then they were electrically conductively processed by coating, in a vacuum, with a thin layer of gold at 30 mA for 80 seconds, using a sputter coater (Hitachi E-1045 ion sputter, Hitachi Ltd., Tokyo, Japan). The surface morphology of the films was observed with the magnification range × 1000–300,000.

### 2.7. The In Vitro Release Profile of Carbendazim from the Composite Films

**Figure 2:**
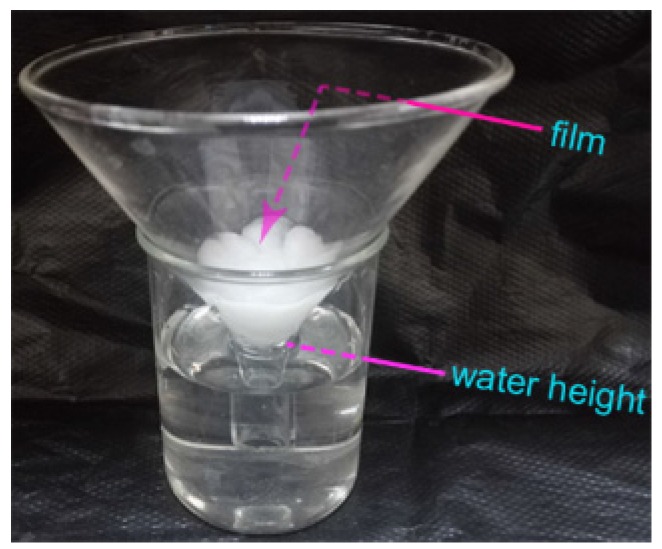
The illustration of the apparatus used in the in vitro release experiments of carbendazim from the composite films.

The chitosan solution with β-CD-EP/carbendazim complexes (carbendazim, 100 μg/mL) was poured into 60 mm diameter glass Petri dishes (volume of casting = 20 mL). The films were dried for 12 h at 45 °C and taken out. To a 100 mm diameter short funnel with 0.3 g cotton on the bottom, the film was placed on the wet cotton (Figure 2). At a fixed time, 5.0 mL of each sample was withdrawn, and was assayed by spectrofluorimetric analysis for the carbendazim concentration. An equal volume of fresh medium was fed back into the solution. All the tests were run in triplicate. The cumulative release fraction (*Q*) of carbendazim was calculated as Equation (4):

(4) $$Q=\frac{C_{0}-C_{t}}{C_{0}}\times 100\%$$

where *C*_0_ is the initial carbendazim content and *C_t_* is the carbendazim content at time *t*.

### 2.8. The In Vivo Efficacy of the Composite Films

**Figure 3:**
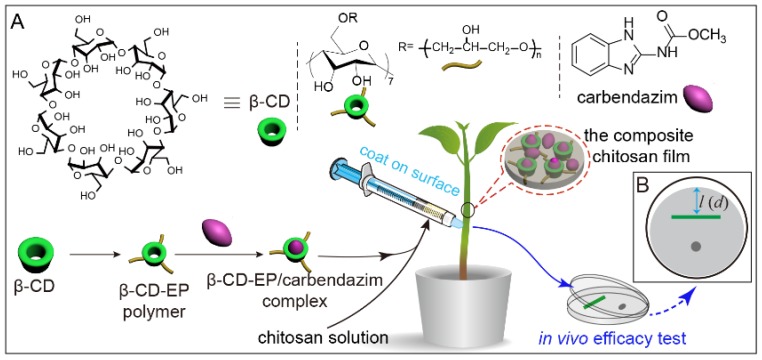
(**A**) the illustration of the preparation of the β-cyclodextrin-epichlorohydrin (β-CD-EP)/carbendazim complex and the in vivo efficacy test; (**B**) the experimental setup of the confrontation assay for preliminarily calculating the mycelial growth inhibition rates (*l* or *d*, the distance of mycelial growth across the stems for control or treatment).

The oilseed rapes used in the in vivo efficacy tests were seedlings that germinated after ten days. An aliquot of 500 μL β-CD-EP/carbendazim-chitosan solution (carbendazim, 100 μg/mL) was withdrawn by a 1.0 mL syringe without a needle. Then the solution was slowly injected in portions and was coated on seedling stems from top to bottom at the same time after the previous coating was dry. The carbendazim solution used as the positive control was at a concentration of 500 μg/mL, which is the minimum concentration recommended to be applied in the field. Ten milliliters of carbendazim solution was sprayed on the stems of 3 seedlings. The untreated controls were sprayed with 10 mL of tap water containing 500 μL chitosan. Three seedlings in one pot comprised one replicate. All the tests were run in triplicate. Five treatments were set to validate the lasting period of carbendazim. All the plants were cultivated in the sterile sandy soil and were placed in a sterile room. The seedlings of each treatment were uprooted at the time of the first, 7th, 14th, and 21st day after the application of carbendazim, respectively. After cutting the roots off, 2 cm of the stem base was cut off and the cutting stems were sterilized by ultraviolet light before incubation. The antifungal efficacy was identified by the plate confrontation assay. On the potato dextrose agar PDA agar plates, the 0.5 mm mycelium disk of *S. sclerotiorum* (Lib.) de Bary was placed on one end and the cutting stem was placed on the other end. After incubation, the Petri dish was inverted and incubated at 25 °C for 48 h (Figure 3). The mycelial growth inhibition rates (*I*) could be preliminarily calculated in a similar manner to Equation (3).

## 3. Results and Discussion

### 3.1. Synthesis and Characterization of β-CD-EP Polymers

The most used and straightforward approach to synthesize β-CD-based polymers is by the reaction of β-CD with EP through the epoxy ring opening polycondensation. This single step polycondensation catalyzed by alkali gave a resulting mixture comprising of monomeric and polymeric units. After successive ultra-filtration and grinding of the mixed product, the final β-CD-EP polymers were obtained in the form of a powder with a particle size of 1–2 mm in diameter. It has been demonstrated that this method produces soluble branched structures of low molecular weight or insoluble polymers when feeding different EP/β-CD ratios [39,40,41]. From our results, the appearance of insoluble polymers was observed for an EP/β-CD ratio higher than 15 when the reaction time was over 2 h. For the soluble samples in our cases, the molecular weights were 1551 and 1756 g/mol for the β-CD-EP-1 and 2 polymers determined by ESI-MS (the most abundant ion peak), suggesting that ours are the monomerically branched β-CD-EP polymers without crosslinking between the β-CDs.

**Figure 4:**
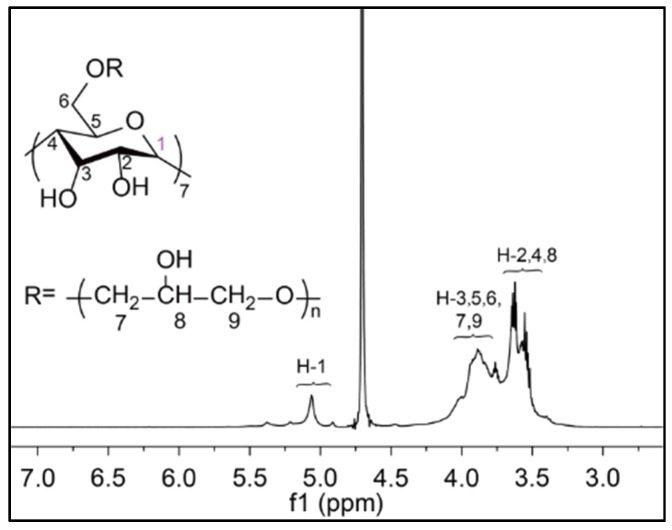
^1^H NMR spectrum of β-CD-EP-2 using D_2_O as a solvent.

**Figure 5:**
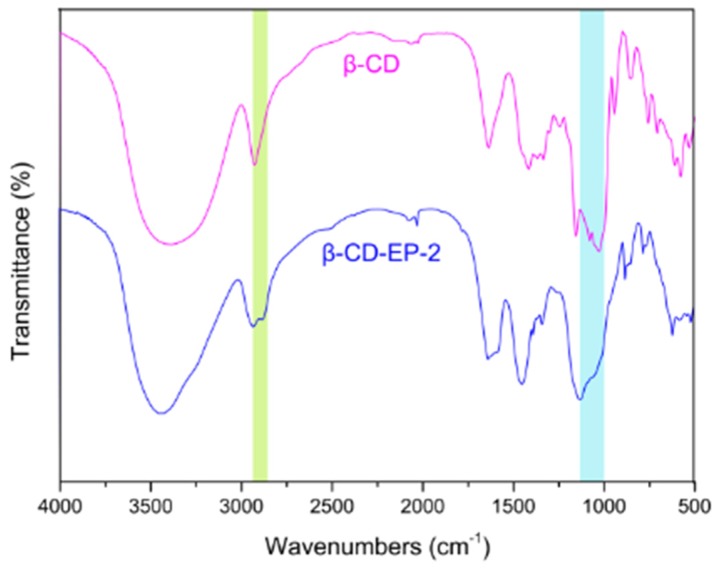
FTIR spectra in the region of 4000–500 cm^−1^ of β-CD and β-CD-EP-2.

The ^1^H NMR spectrum was used to identify the polymeric products and their β-CD content. As can be seen from Figure 4, there are two broadened peaks between 3.3 and 4.5 ppm, which are assigned to protons from C-7, 8, and 9 of the branched chains, and C-2, 3, 4, 5, and 6 of the β-CD pyranose rings, respectively [31]. A small single peak around 5.0 ppm belonged to the C-l proton of the β-CD glucoses [31]. Each EP molecule after incorporation into β-CD led to a five-hydrogen increment, which could be calculated from the integration value of the hydrogen signals between 3.3 and 4.5 ppm [31]. Thus the β-CD content in the β-CD-EP polymers could be determined through the ratio of the peak integration. When the β-CD/EP feeding ratio is increased from 1/15 to 1/8, the CD content increases from 440 mg/g to 750 mg/g accordingly (Table 1). The FT-IR spectra of the branched β-CD-EP polymers showed the typical changes of the four characteristic bands which were different from that of β-CD (Figure 5). The stretching vibration bond of C-C (1038 cm^−1^) and C-O (1091 cm^−1^) were enhanced in the β-CD-EP polymer as well as the C-O-C asymmetric stretching vibration (1164 cm^−1^). There was also an increase in the asymmetric stretching vibration of CH_2_ at 2876 cm^−1^. These typical changes have been reportedly used to verify the reaction between β-CD and EP [40].

**Figure 6:**
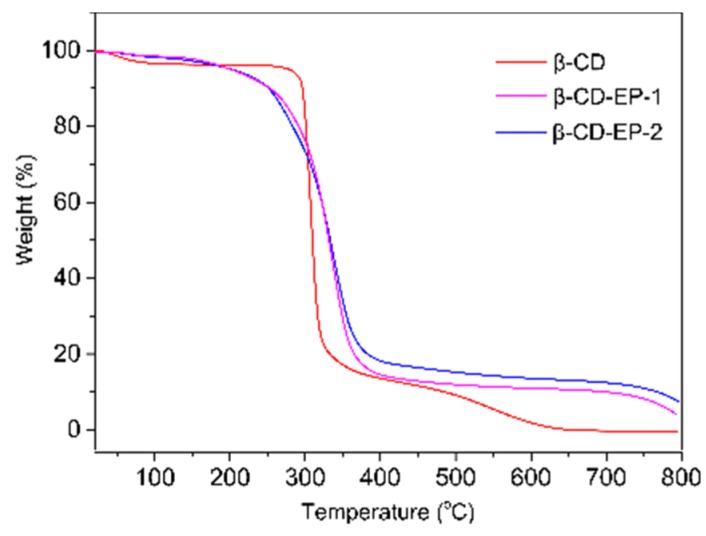
Thermogravimetric curves of β-CD, and β-CD-EP-1 and 2.

To investigate the thermal stability of the β-CD-EP polymers, TGA curves of the prepared samples were scrutinized and shown in Figure 6. As can be seen, the initial temperature of thermal degradation for β-CD occurred at 301 °C, although there was a little weight loss around 2% below 100 °C due to the withdrawal of physically adsorbed water. For β-CD, the pyrolysis temperature of the main weight loss ranged from 301 °C to 311 °C. Eventually, β-CD loses up to 98% of its total weight at 700 °C, which could presumably be due to the pyrolysis of almost all of the fundamental backbone of the polymer at such a high temperature. On the other hand, the onset temperature of thermal degradation for the β-CD-EP polymers slightly decreased to 280 °C. For both the β-CD-EP-1 and 2 samples, the pyrolysis temperature of the main weight loss ranged from 280 to 370 °C. The increased upper pyrolysis temperature of the main weight loss could be attributed to the chemical grafting of the poly(2-hydroxypropyl) branched segments onto the β-CD. It was worth noting that due to the presence of the poly(2-hydroxypropyl) branched segments, the β-CD-EP polymers yielded a higher amount of residues more than 10% of their total weight at 700 °C, which was in conjunction with a previously reported study [42].

**Figure 7:**
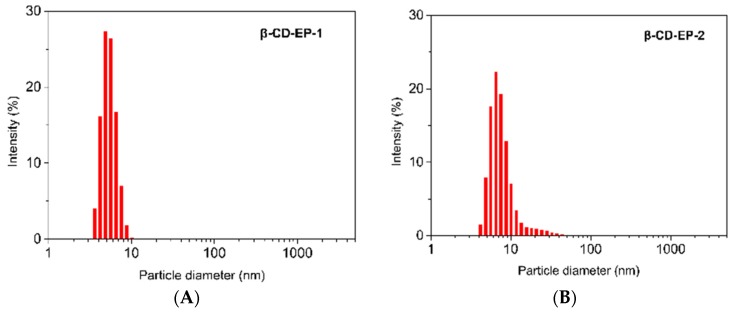
Particle size distributions of the β-CD-EP-1(**A**) and β-CD-EP-2 (**B**) in distilled water.

Figure 7 and Table 1 show the particle size distributions of the β-CD-EP polymers in a dilute solution. Due to the aggregation of polymer particles in a concentrated solution, a dilute solution with a polymer concentration of 5.0 mg/mL was utilized. The average hydrodynamic diameter of β-CD-EP-1 and 2 measured by the DLS equipment at a 90° angle were 5.2 ± 0.5 nm and 8.3 ± 1.3 nm, respectively. The solubilities of β-CD-EP-1 and 2 were 35.4 ± 1.6 g/100 mL and 42.3 ± 1.5 g/100 mL, whose β-CD solubilities are 14.3 and 9.8 times higher than that of the β-CD (1.8 g/100 mL), respectively. The reason for the low aqueous solubility of β-CD is that the hydrogen bonding that exists intermolecularly between the secondary hydroxyl groups shields the interactions of β-CD and its surrounding water molecules. Accordingly, only by disruption of the intermolecular hydrogen bonding can its solubility be enhanced. Although the EP content in the polymer increases from 250 mg/g in β-CD-EP-1 to 560 mg/g in β-CD-EP-2, the solubility changed marginally, suggesting that increasing the polymerization degree is not indispensable for further disruption of the original crystallinity of β-CD. Meanwhile, the good water solubility of the β-CD-EP polymers once again confirmed that intermolecular and intramolecular crosslinking did not occur.

### 3.2. Interactions between β-CD-EP and Carbendazim in Solution

#### 3.2.1. Spectrofluorimetric Analysis of Carbendazim

**Figure 8:**
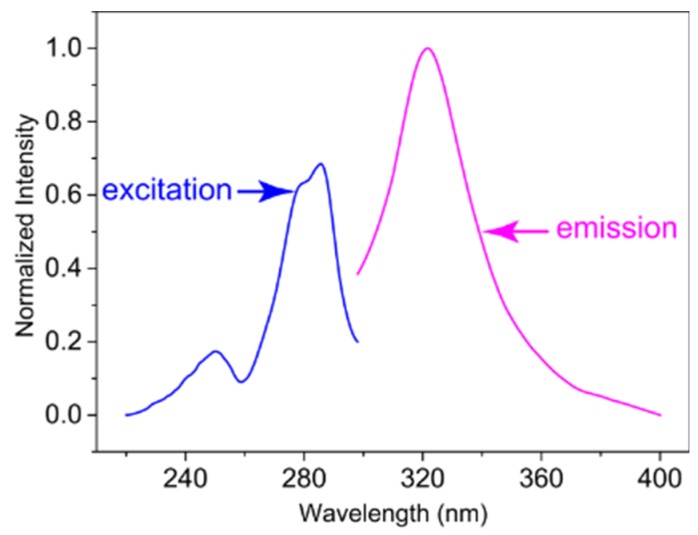
The normalized fluorescence excitation (**1**) and emission (**2**) spectra of carbendazim (0.025 mmol/L) in distilled water (λ_ex_ = 285 nm, λ_em_ = 320 nm).

Normalized fluorescence spectra of carbendazim in excitation and emission are shown in Figure 8. The maximum wavelength of the excitation (λ_ex_) and emission (λ_em_) occurred at 285 nm and 320 nm, respectively. The data were recorded at 320 nm (λ_ex_ = 285 nm) for different concentrations of carbendazim and the linear regression equation was *F* = 4.341*C* + 10.867 (*r* = 0.985), 0.01 mmol/L ≤ *C* ≤ 0.04 mmol/L.

#### 3.2.2. Phase Solubility Studies of the β-CD-EP/Carbendazim Complexes

**Figure 9:**
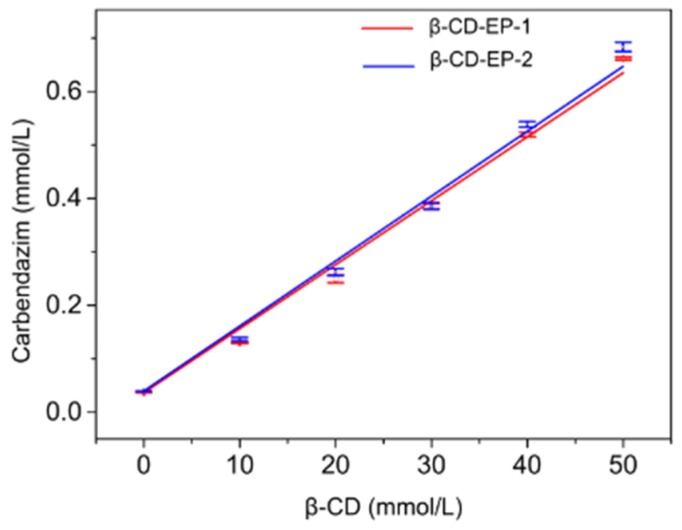
Phase-solubility diagrams of carbendazim in aqueous β-CD-EP-1 and 2.

The phase solubility diagrams of carbendazim in aqueous β-CD-EP-1 and 2 solutions are shown in Figure 9. As can be seen, the water solubility of carbendazim linearly increased as a function with the β-CD concentration. The solubility of the poorly soluble carbendazim (ca. 0.0367 mmol/L) could be significantly enhanced through complexation. For example, at the β-CD concentration of 50 mmol/L, the water solubility of carbendazim increased to 0.662 mmol/L for β-CD-EP-1 and 0.680 mmol/L for β-CD-EP-2—almost 17.9 and 18.5 times higher than that in the aqueous solution, respectively. The apparent solubilizing efficiency of the β-CD-EP polymer was higher than that of the hydroxypropyl-β-CD at 50 mmol/L, which reportedly afforded a 4.2-fold increase in the solubility of carbendazim. According to the literature, the phase solubility diagram of carbendazim was the A_L_ type, indicating that a 1:1 inclusion complex between carbendazim and the β-CD-EP polymers was formed [35]. The values of *K_s_*, *r*^2^, and slope for the phase solubility study in distilled water are summarized in Table 2. When compared, the *K_s_* of β-CD-EP-2 (334.8 M^−1^) was marginally higher than that of β-CD-EP-1 (330.1 M^−1^), which may be ascribed to the weak interactions of carbendazim with the more branched polymeric chains of β-CD-EP-2. Considering that β-CD-EP-2 contributed to the superior solubilizing efficiency over β-CD-EP-1, the β-CD-EP-2 polymeric system was chosen in the following studies.

#### 3.2.3. Determination of Complex Stoichiometry

**Figure 10:**
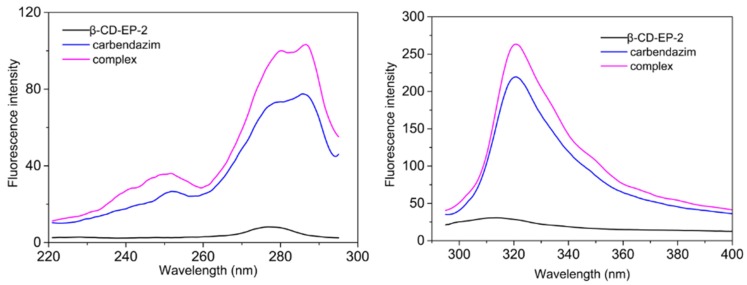
The fluorescence spectra of 0.6 mmol/L β-CD-EP-2, 0.6 mmol/L carbendazim, and 0.6 mmol/L β-CD-EP-2 + 0.6 mmol/L carbendazim (λ_ex_ = 285 nm, λ_em_ = 320 nm).

Figure 10 showed the fluorescence spectra of carbendazim aqueous solutions in the absence and presence of the β-CD-EP polymer. The addition of β-CD-EP led to a slight increase in the fluorescence intensity below 310 nm, whereas an obvious increase was observed above 310 nm. The increase in fluorescence intensity revealed the inclusion complex formation between carbendazim and β-CD. This fluorescence intensity increase caused by β-CD could be ascribed to the guest molecule carbendazim, without geometrical change occurring in a twisted intramolecular charge-transfer excited-state and the elimination of fluorescence quenching generated by the oxygen in aqueous solution [43,44].

**Figure 11:**
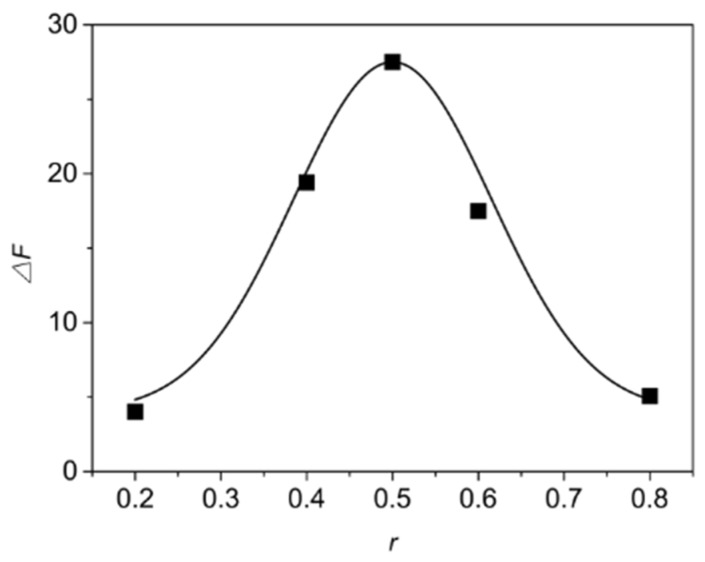
Continuous variation plot (Job’s plot) for the β-CD-EP-2/carbendazim complex.

The Job’s method to determine the complex stoichiometry was performed by the continuous variation plot. This method utilizes an indicating parameter (*△F*) which is directly associated to the concentration of the inclusion complex in the aqueous solution. This indicating parameter was then plotted versus the molar fraction of carbendazim (*r*) (*r* = [carbendazim]/[carbendazim] + [β-CD-EP]). The stoichiometry of the complex could be determined according to the *r* position corresponding to the maximum value of *△F*; for example, the *r* position around 0.3 indicated a 2:1 stoichiometry of the host-guest, while 0.5 indicated a 1:1 stoichiometry. As illustrated in Figure 11, the *r* position corresponding to the peak of *△F* appeared at 0.5, implying a 1:1 complex stoichiometry of carbendazim and the β-CD-EP polymer. An explanatory reason for this is that the adjacent intermolecular β-CD units of the β-CD-EP-2 polymer are not inclined to include the same guest molecule since such inclination could be hindered from the branched polymeric chains. In addition, the continuous variation plot was not influenced by the branched chains, although they may provide carbendazim with non-inclusion interactions.

### 3.3. The In Vitro Antifungal Effects of β-CD-EP/Carbendazim Complexes

**Figure 12:**
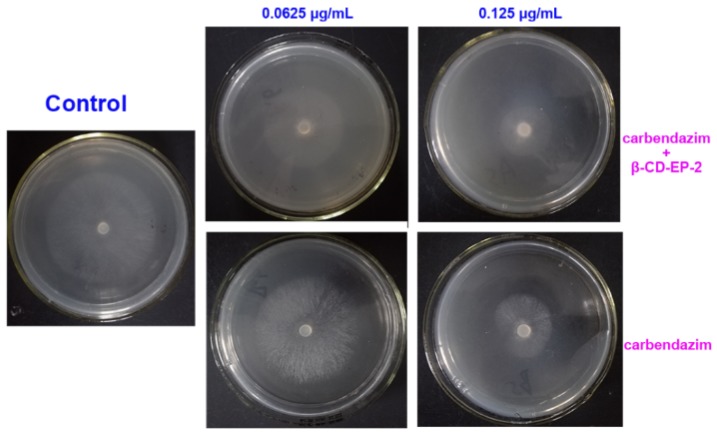
The in vitro antifungal effects of carbendazim and β-CD-EP-2/carbendazim on *S. sclerotiorum* (Lib.) de Bary. For the control, the mycelial growth diameter was 70.0 ± 1.0 mm; at 0.0625 μg/mL of carbendazim, the mycelial growth diameters were 58.0 ± 1.8 mm for carbendazim and 54.1 ± 1.6 mm for the β-CD-EP/carbendazim complex; at 0.125 μg/mL, the mycelial growth diameters were 30.6 ± 2.1 mm for carbendazim and 23.5 ± 1.6 mm for the β-CD-EP/carbendazim complex.

In the mycelial growth inhibition assay, it was found that the β-CD-EP/carbendazim complex could significantly inhibit the mycelial growth against *S. sclerotiorum* (Lib.) de Bary rather than the carbendazim used alone (Figure 12), indicating that the inclusion of carbendazim into the β-CD-EP polymer improved the bioavailability of carbendazim. The antifungal activity in Table 3 revealed that the EC_50_ value of carbendazim against *S. sclerotiorum* (Lib.) de Bary was 0.103 μg/mL, whereas the EC_50_ of the β-CD-EP/carbendazim complex was 0.0885 μg/mL. As compared with the antifungal activity of carbendazim used alone, there was a slight increase in the efficiency of the β-CD-EP/carbendazim complex. Similar results have also been elaborated by Boldescu et al. [45,46]. Since the β-CD has the complex ability to solubilize some biomacromolecules on the membrane, such as proteins, cholesterol, and lipids, it was supposed that this complexation increases the permeability of the microbial cell and readily promotes the penetration of antimicrobial agents [47].

### 3.4. Characterization of Chitosan and β-CD-EP/Carbendazim Composite Films

**Figure 13:**
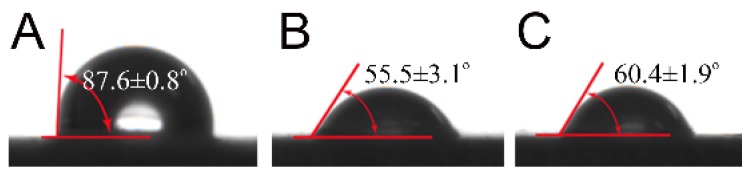
Surface contact angles. (**A**) Chitosan film; (**B**) Chitosan and β-CD-EP-2 composite film; (**C**) Chitosan and β-CD-EP-2/carbendazim composite film.

**Figure 14:**
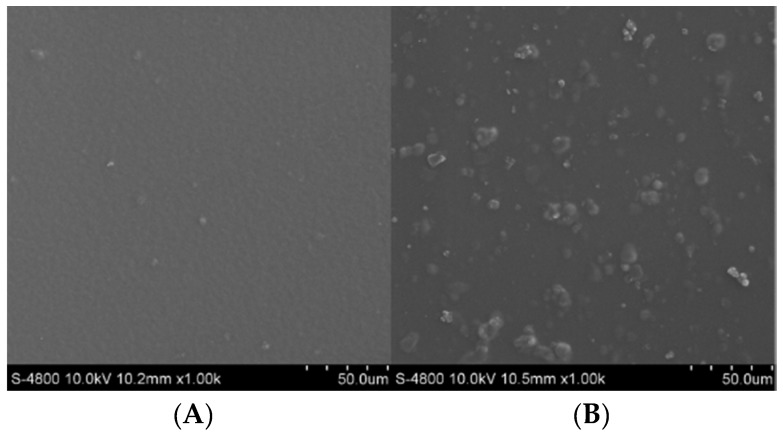
SEM micrographs of the chitosan film (**A**), and the chitosan and β-CD-EP-2/carbendazim composite film (**B**).

The physical and surface properties of the chitosan and β-CD-EP-2/carbendazim composite films were explored through static contact angle studies and SEM, respectively. The contact angle of the pure chitosan film exhibited a value of 87.6 ± 0.8° (Figure 13A), while the angle decreased to 55.5 ± 3.1° after composition of the chitosan film with the β-CD-EP polymer (Figure 13B). When the film was composed with the β-CD-EP/carbendazim complex, the angle changed slightly and was 60.4 ± 1.9° (Figure 13C). It has been reported that the surface properties of the chitosan film could be fine-tuned by the composition of different materials; for instance, when pectin was attached to the chitosan films, they became more hydrophobic [48]. As in our case, the results indicated that the composition of the hydrophilic β-CD-EP polymer resulted in more hydrophilic chitosan films. It should be noted that this hydrophilicity might be unfavourable for applying this composite film in such rainy or moist circumstances, which might shorten its effective duration. The SEM images of the chitosan film and composite film are presented in Figure 14. The chitosan film was found to be smooth and uniform. After composition of the chitosan film with the β-CD-EP polymer (data not shown) or the β-CD-EP/carbendazim complex, there were many particles in the films or on the surface of the films, which might be due to the aggregation of the β-CD-EP polymer that lowers the surface energy in the curing process of the chitosan film.

### 3.5. The In Vitro Release Profile of Carbendazim from the Composite Films

The chitosan film has been widely applied as an emerging platform in drug controlled release systems due to its nontoxicity, biocompatibility, and biodegradability [49]. These properties have also attracted increasing attention due to their potential applications in the production of edible films composited with bioactive ingredients in the fields of food processing or packaging [50,51]. The carbendazim release behaviors from the chitosan and β-CD-EP-2/carbendazim composite film were performed according to the above designed method (Section 2.7) to simulate the actual situation.

**Figure 15:**
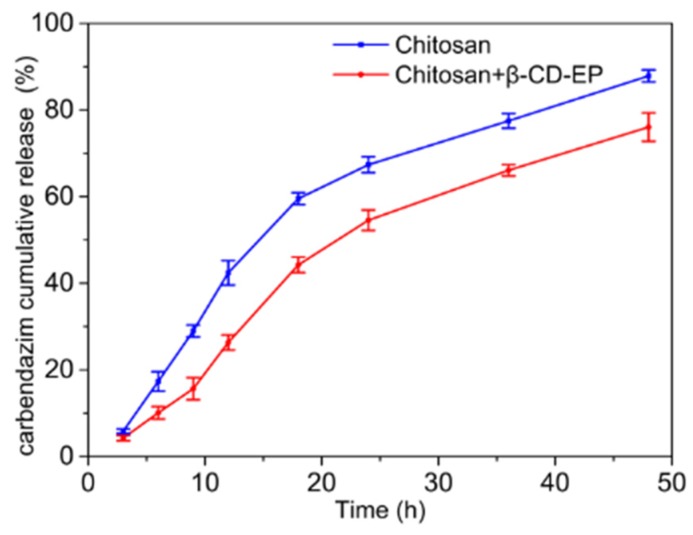
The release profiles of carbendazim from the chitosan film and the chitosan and β-CD-EP-2/carbendazim composite film.

The release profiles of carbendazim from the chitosan films and the chitosan and β-CD-EP-2 composited films are shown in Figure 15. An obvious burst release of the carbendazim was observed from 3 h to 18 h for the chitosan films and from 9 h to 18 h for the chitosan and β-CD-EP-2 composited films, being longer in the case of the non-containing β-CD-EP-2 films (ca. 15 h vs. 9 h). In addition, an obvious retard of release for the chitosan and β-CD-EP-2 composite films was achieved in the initial 9 h. After 48 h, around 87% and 76% of carbendazim was released from the chitosan films and the chitosan and β-CD-EP-2 composited films, respectively. The release profiles of the above systems during a certain period could be described by the Higuchi kinetics, a square root of time kinetics: *Q = kt*^1/2^, where *Q* is the cumulative percentage of the drug released at a given time *t*, and *k* is the Higuchi dissolution constant [52]. The release data of the chitosan films and the chitosan and β-CD-EP-2 composited films could be well fitted with the equations *Q*_1_ *= 12.9 t*^1/2^ (3 h ≤ *t* *≤* 48 h) and *Q*_2_ *= 10.8 t*^1/2^ (9 h ≤ *t ≤* 48 h), respectively. Since the Higuchi kinetic model describes drug release as a diffusion process rooted in Fick’s law, the lower Higuchi dissolution constant (*k*) of the chitosan and β-CD-EP-2 composite films implied higher diffusional barriers when carbendazim was co-immobilized in this system, leading to a preferable controlled release profile when compared with the chitosan film system [52].

The polymeric matrix has been popularly used in many dosage formulations to achieve drug-controlled delivery [53]. For example, Bai et al. have reported a monolithic starch-based hydrogel that can load the carbendazim and control its release profile used in soil [54]. Sandhya et al. and Campos et al. have prepared polymeric nanoparticles to achieve the controlled release characteristics of carbendazim while enhancing its chemical stability [55,56]. These polymeric formulations, however, were mostly scheduled to be applied in soil or aerial spraying. It has been demonstrated that excipients such as β-CDs could efficiently modulate the drug release profiles [57]. In this work, β-CD-EP polymers were proven to be effective in modulating drug release behaviors from the polymeric film, such as the release retard and rate. Further studies will focus on detailed investigations of the structure-performance relationship of β-CD-EP in controlled release systems for different pesticides.

### 3.6. The In Vivo Efficacy of the Composite Films

**Figure 16:**
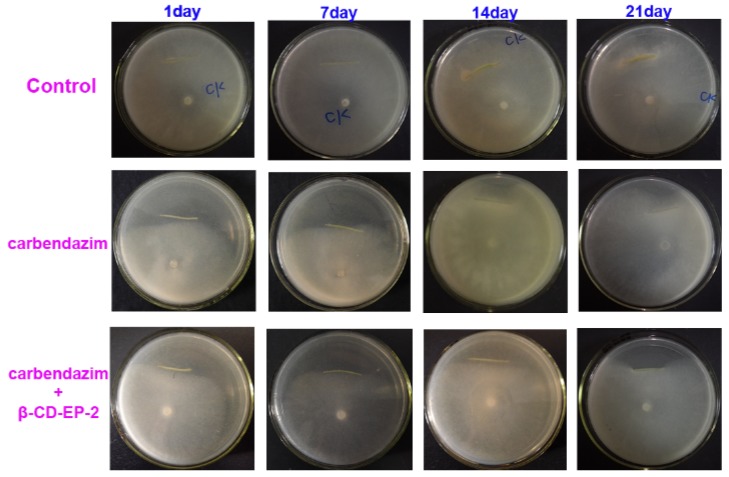
The in vivo efficacies of carbendazim and the chitosan and β-CD-EP-2/carbendazim composite film.

The potential of the chitosan and β-CD-EP-2/carbendazim composite film used in vivo as a controlled release system to protect oilseed rape against *S. sclerotiorum* (Lib.) de Bary was evaluated. At the tenth day after germination of the oilseed rape seeds, overground stems of seedlings were coated with the carbendazim-loaded composite films (Figure 3) or were sprayed with the carbendazim solutions. It should be noted that the recommended effective concentration of carbendazim solutions used in field to control *S. sclerotiorum* (Lib.) de Bary is higher than 500 μg/mL. In this study, we sprayed the carbendazim at a concentration of 500 μg/mL, while the concentration of carbendazim in the composited film was 100 μg/mL. The antifungal efficacies of sprayed or coated treatments were assessed after 1 day, 7 days, 14 days, and 21 days by the plate confrontation assay. The results of the plate confrontation assay are shown in Figure 16.

As shown in Figure 16 and Table 4, the antifungal efficacy was identified by inhibiting mycelial growth across the stems. For the controls, the mycelia of *S. sclerotiorum* (Lib.) de Bary could grow across the stems and were spread identically over the surface of the PDA culture medium. When treated with carbendazim, the stems kept a complete antifungal efficiency (100%) after 7 days while reducing their efficacy after 14 days (52.7%) and after 21 days (28.2%). However, as compared with those treated with carbendazim alone, the stems coated with the chitosan and β-CD-EP/carbendazim composited films remained effective for a longer period, 21 days. After that (at 28 days), the efficacy was clearly decreased to 49.4%. 

As discussed above, β-CDs could solubilize the guest molecules and have been used in sustained release platforms with the aim of tuning the drug release profile. Due to the complex formation between β-CDs and guest molecules, β-CDs can protect guest molecules against decomposition reactions induced by physical (light or heat), chemical (oxidation or hydrolysis), and biodegradable actions [58,59]. Meanwhile, a synergism was observed for the β-CD-EP/carbendazim complex. Considering these advantages, β-CD-EP, therefore, could offer the added bonus of remaining effective for longer durations. It should be noted that the decrease in the applied amount of carbendazim together with the controlled-release property could reduce the harmful impact of fungicides on the environment that was caused by the drift effect. This work provided a new strategic insight in the control of plant diseases by mean of a plant healthcare material—the chitosan and polymeric β-CD composite film. In our following works, we will extend this plant healthcare material not only in the control of crop diseases but also in ornamental plant and fruit tree disease control.

## 4. Conclusions

In this work, we first prepared and characterized the β-CD-EPs polymers. Then the interaction between the β-CD-EP and carbendazim was investigated. The results indicated that β-CD-EP could significantly improve the solubility of the guest molecule carbendazim and the stoichiometry with a host-guest ratio of 1:1 was confirmed by the Job’s plot. A slight synergism was observed for the β-CD-EP-2/carbendazim complex, indicating an enhancement to the bioavailability of carbendazim. Meanwhile, in vitro release studies revealed that β-CD-EP could effectively modulate carbendazim release behaviors, such as the release retard and rate. The in vivo efficacy experiments demonstrated the β-CD-EP could offer the added bonus of remaining effective for longer durations. Overall, chitosan and the β-CD-EP polymer composite film as a plant healthcare material for a controlled release system could decrease the applied amount of carbendazim and thus reduce the harmful impact on the environment that is caused by drift effect. This work provided a new strategic insight in the control of plant diseases by mean of a plant healthcare material—the chitosan and polymeric β-CD-EP composite film. In our following works, we will extend this plant healthcare material not only in the control of crop diseases but also in the control of ornamental plant and fruit tree diseases.

## Figures and Tables

**Table 1:** Experimental conditions and properties of the β-CD-EP polymers.

Sample	EP/β-CD (Molar Ratio)	Time of Gelation (h)	β-CD Content (mg/g)	Hydrodynamic Diameter (nm)	Solubility (g/100 mL)
β-CD-EP-1	8/1	-	750	5.2 ± 0.5	35.4 ± 1.6
β-CD-EP-2	15/1	-	440	8.3 ± 1.3	42.3 ± 1.5
β-CD-EP-3	17/1	2 h	-	-	-

**Table 2:** The values of *K_s_*, *r*^2^, and the slope of β-CD-EP-1 and 2 for the phase solubility study, and the solubilizing efficiency and LE (%) of β-CD-EP-1 and 2.

Sample	Slope	*r*^2^	*K_s_* (M^−1^)	Solubilizing Efficiency	LE (%)
β-CD-EP-1	0.01197	0.9965	330.1	17.9 ± 0.2	27.4 ± 2.1
β-CD-EP-2	0.01214	0.9997	334.8	18.5 ± 0.3	31.2 ± 1.6

**Table 3:** The toxicity equations and EC_50_ of carbendazim and β-CD-EP-2/carbendazim against *S. sclerotiorum* (Lib.) de Bary.

System	Toxicity Regression Equation	Correlation Coefficient (*r*)	EC_50_ (μg/mL)	Synergistic Times
carbendazim	y = 8.397 + 3.438x	0.9928	0.103	-
β-CD-EP-2/carbendazim	y = 8.695 + 3.508x	0.9959	0.0885	1.16

**Table 4:** The mycelial growth inhibition rates (*I*) of carbendazim and the chitosan and β-CD-EP-2/carbendazim composite film in the in vivo efficacy test at different days.

System	The Mycelial Growth Inhibition Rates (%)				
	1 day	7 days	14 days	21 days	28 days
carbendazim	100	100	52.7 ± 3.4	28.2 ± 9.5	-
β-CD-EP-2/carbendazim	100	100	100	100	49.4 ± 5.6

## References

[1] Anderson P.K., Cunningham A.A., Patel N.G., Morales F.J., Epstein P.R., Daszak P. (2004). Emerging infectious diseases of plants: Pathogen pollution, climate change and agrotechnology drivers. Trends Ecol. Evol..

[2] Fisher M.C., Henk D.A., Briggs C.J., Brownstein J.S., Madoff L.C., McCraw S.L., Gurr S.J. (2012). Emerging fungal threats to animal, plant and ecosystem health. Nature.

[3] Vurro M., Bonciani B., Vannacci G. (2010). Emerging infectious diseases of crop plants in developing countries: Impact on agriculture and socio-economic consequences. Food Secur..

[4] Wagacha J.M., Muthomi J.W. (2008). Mycotoxin problem in Africa: Current status, implications to food safety and health and possible management strategies. Int. J. Food Microbiol..

[5] Godfray H.C.J., Beddington J.R., Crute I.R., Haddad L., Lawrence D., Muir J.F., Pretty J., Robinson S., Thomas S.M., Toulmin C. (2010). Food Security: The Challenge of Feeding 9 Billion People. Science.

[6] Seaman D. (1990). Trends in the formulation of pesticides—An overview. Pestic Sci..

[7] Longley M., Sotherton N.W. (1997). Factors determining the effects of pesticides upon butterflies inhabiting arable farmland. Agric. Ecosyst. Environ..

[8] de Snoo G.R., van der Poll R.J. (1999). Effect of herbicide drift on adjacent boundary vegetation. Agric. Ecosyst. Environ..

[9] de Snoo G.R., de Wit P.J. (1998). Buffer Zones for Reducing Pesticide Drift to Ditches and Risks to Aquatic Organisms. Ecotox Environ. Safe.

[10] Bahadir M., Pfister G., Haug G., Hoffmann H. (1990). Controlled Release Formulations of Pesticides. Controlled Release, Biochemical Effects of Pesticides, Inhibition of Plant Pathogenic Fungi.

[11] Biwer A., Antranikian G., Heinzle E. (2002). Enzymatic production of cyclodextrins. Appl. Microbiol. Biot..

[12] Schöffer J.d.N., Klein M.P., Rodrigues R.C., Hertz P.F. (2013). Continuous production of β-cyclodextrin from starch by highly stable cyclodextrin glycosyltransferase immobilized on chitosan. Carbohydr. Polym..

[13] Szejtli J. (1998). Introduction and General Overview of Cyclodextrin Chemistry. Chem. Rev..

[14] Qian L., Guan Y., Xiao H. (2008). Preparation and characterization of inclusion complexes of a cationic β-cyclodextrin polymer with butylparaben or triclosan. Int. J. Pharm..

[15] Daoud-Mahammed S., Ringard-Lefebvre C., Razzouq N., Rosilio V., Gillet B., Couvreur P., Amiel C., Gref R. (2007). Spontaneous association of hydrophobized dextran and poly-β-cyclodextrin into nanoassemblies.: Formation and interaction with a hydrophobic drug. J. Colloid Interface Sci..

[16] Nie S., Zhang S., Pan W., Liu Y. (2011). *In vitro* and *in vivo* studies on the complexes of glipizide with water-soluble β-cyclodextrin–epichlorohydrin polymers. Drug Dev. Ind. Pharm..

[17] He X., Li K., Xing R., Liu S., Hu L., Li P. (2016). The production of fully deacetylated chitosan by compression method. Egypt. J. Aquat. Res..

[18] Younes I., Rinaudo M. (2015). Chitin and Chitosan Preparation from Marine Sources. Structure, Properties and Applications. Mar. Drugs.

[19] van de Manakker F., Vermonden T., van Nostrum C.F., Hennink W.E. (2009). Cyclodextrin-Based Polymeric Materials: Synthesis, Properties, and Pharmaceutical/Biomedical Applications. Biomacromolecules.

[20] van Haver E., Alink G.M., Cockburn A., Kuiper H.A., Peijnenburg A.A.C.M. (2008). Safety and nutritional assessment of GM plants and derived food and feed: The role of animal feeding trials. Food Chem. Toxicol..

[21] Stephenson A.L., Dennis J.S., Scott S.A. (2008). Improving the sustainability of the production of biodiesel from oilseed rape in the UK. Process Saf. Environ. Prot..

[22] Twengström E., Sigvald R., Svensson C., Yuen J. (1998). Forecasting Sclerotinia stem rot in spring sown oilseed rape. Crop Prot.

[23] Peltier A.J., Bradley C.A., Chilvers M.I., Malvick D.K., Mueller D.S., Wise K.A., Esker P.D. (2012). Biology, Yield loss and Control of Sclerotinia Stem Rot of Soybean. J. Integr. Pest Manag..

[24] Derbyshire M.C., Denton-Giles M. (2016). The control of sclerotinia stem rot on oilseed rape (Brassica napus): Current practices and future opportunities. Plant Pathol..

[25] Stephen-Haynes J., Callaghan R., Wibaux A., Johnson P., Carty N. (2014). Clinical evaluation of a thin absorbent skin adhesive dressing for wound management. J. Wound Care.

[26] Matsumura H., Imai R., Ahmatjan N., Ida Y., Gondo M., Shibata D., Wanatabe K. (2014). Removal of adhesive wound dressing and its effects on the stratum corneum of the skin: Comparison of eight different adhesive wound dressings. Int. Wound J..

[27] Quinn J., Lowe L., Mertz M. (2000). The Effect of a New Tissue-Adhesive Wound Dressing on the Healing of Traumatic Abrasions. Dermatology.

[28] Boateng J.S., Matthews K.H., Stevens H.N. E., Eccleston G.M. (2008). Wound Healing Dressings and Drug Delivery Systems: A Review. J. Pharm. Sci..

[29] Khan T.A., Peh K.K., Ch'Ng H.S. (2002). Reporting degree of deacetylation values of chitosan: The influence of analytical methods. J. Pharm. Pharm. Sci..

[30] Knaul J.Z., Kasaai M.R., Bui V.T., Creber K.A.M. (1998). Characterization of deacetylated chitosan and chitosan molecular weight review. Can. J. Chem..

[31] Renard E., Deratani A., Volet G., Sebille B. (1997). Preparation and characterization of water soluble high molecular weight β-cyclodextrin-epichlorohydrin polymers. Eur. Polym. J..

[32] Xin J., Guo Z., Chen X., Jiang W., Li J., Li M. (2010). Study of branched cationic β-cyclodextrin polymer/indomethacin complex and its release profile from alginate hydrogel. Int. J. Pharm..

[33] Maddens T., Vélaz I., Machín R., Isasi J.R., Martín C., Martínez-Ohárriz M.C., Zornoza A. (2011). Complexation of ebastine with β-cyclodextrin derivatives. J. Incl. Phenom. Macrocycl. Chem..

[34] Mura P., Faucci M.T., Maestrelli F., Furlanetto S., Pinzauti S. (2002). Characterization of physicochemical properties of naproxen systems with amorphous β-cyclodextrin-epichlorohydrin polymers. J. Pharm. Biomed. Anal..

[35] Higuchi T.A., Connors K.A. (1965). Phase-Solubility Techniques. Advances in Analytical Chemistry and Instrumentation.

[36] Job P. (1928). Formation and stability of inorganic complexes in solution. Ann. Chim.-Rome.

[37] Renny J.S., Tomasevich L.L., Tallmadge E.H., Collum D.B. (2013). Method of Continuous Variations: Applications of Job Plots to the Study of Molecular Associations in Organometallic Chemistry. Angew. Chem. Int. Edit..

[38] Zhu X.-L., Wang H.-B., Chen Q., Yang W.-C., Yang G.-F. (2007). Preparation and Characterization of Inclusion Complex of Iprodione and β-Cyclodextrin to Improve Fungicidal Activity. J. Agric. Food Chem..

[39] Sainz-Rozas P.R., Isasi J.R., González-Gaitano G. (2005). Binding of dibenzofuran and its derivatives to water-soluble β-cyclodextrin polymers. J. Photochem. Photobiol. A: Chem..

[40] Gómez-Galván F., Pérez-Álvarez L., Matas J., Álvarez-Bautista A., Poejo J., Duarte C.M., Ruiz-Rubio L., Vila-Vilela J.L., León L.M. (2016). Preparation and characterization of soluble branched ionic β-cyclodextrins and their inclusion complexes with triclosan. Carbohydr. Polym..

[41] Ao X., Stenken J.A. (2003). Water-soluble cyclodextrin polymers for enhanced relative recovery of hydrophobic analytes during microdialysis sampling. Analyst.

[42] Pham T.A., Kumar N.A., Jeong Y.T. (2010). Covalent functionalization of graphene oxide with polyglycerol and their use as templates for anchoring magnetic nanoparticles. Synth. Met..

[43] Abdel-Shafi A.A. (2007). Inclusion complex of 2-naphthylamine-6-sulfonate with β-cyclodextrin: Intramolecular charge transfer versus hydrogen bonding effects. Spectrochim. Acta A: Mol. Biomol. Spectrosc..

[44] Du X.Z., Zhang Y., Jiang Y.B., Huang X.Z., Chen G.Z. (1997). Study of room-temperature phosphorescence of 1-bromonaphthalene in sodium dodecylbenzene sulfonate and β-cyclodextrin solution. Spectrochim. Acta A: Mol. Biomol. Spectrosc..

[45] Boldescu V., Macaev F., Duca G. (2014). Role of cyclodextrins in new antimycobacterial formulations. Chem. J. Mold..

[46] Fliur M., Veaceslav B., Athina G., Natalia S. (2013). Recent Advances in the Use of Cyclodextrins in Antifungal Formulations. Curr. Top. Med. Chem..

[47] Huang Z., London E. (2013). Effect of Cyclodextrin and Membrane Lipid Structure upon Cyclodextrin–Lipid Interaction. Langmuir.

[48] Croisier F., Jérôme C. (2013). Chitosan-based biomaterials for tissue engineering. Eur. Polym. J..

[49] Luciano N.M., Ignacio M.H., Julio A.L. (2012). Recent Advances in Chitosan Films for Controlled Release of Drugs. Recent Patents Drug Deliv. Formul..

[50] Elsabee M.Z., Abdou E.S. (2013). Chitosan based edible films and coatings: A review. Mater. Sci. Eng.: C.

[51] Aider M. (2010). Chitosan application for active bio-based films production and potential in the food industry: Review. LWT—Food Sci. Technol..

[52] Costa P., Sousa Lobo J.M. (2001). Modeling and comparison of dissolution profiles. Eur. J. Pharm. Sci..

[53] Arifin D.Y., Lee L.Y., Wang C.-H. (2006). Mathematical modeling and simulation of drug release from microspheres: Implications to drug delivery systems. Adv. Drug Deliv. Rev..

[54] Bai C., Zhang S., Huang L., Wang H., Wang W., Ye Q. (2015). Starch-based hydrogel loading with carbendazim for controlled-release and water absorption. Carbohydr. Polym..

[55] Sandhya, Kumar S., Kumar D., Dilbaghi N. (2017). Preparation, characterization, and bio-efficacy evaluation of controlled release carbendazim-loaded polymeric nanoparticles. Environ. Sci. Pollut. Res..

[56] Campos E.V.R., Oliveira J.L.D., da Silva C.M.G., Pascoli M., Pasquoto T., Lima R., Abhilash P.C., Fernandes Fraceto L. (2015). Polymeric and Solid Lipid Nanoparticles for Sustained Release of Carbendazim and Tebuconazole in Agricultural Applications. Sci. Rep..

[57] Sangalli M.E., Zema L., Maroni A., Foppoli A., Giordano F., Gazzaniga A. (2001). Influence of betacyclodextrin on the release of poorly soluble drugs from inert and hydrophilic heterogeneous polymeric matrices. Biomaterials.

[58] Buschmann H.-J., Schollmeyer E. (2002). Applications of cyclodextrins in cosmetic products: A review. J. Cosmet. Sci..

[59] Del Valle E.M.M. (2004). Cyclodextrins and their uses: A review. Process Biochem..

